# Custom Triflange Acetabular and Femoral Components for Primary Hip Arthroplasty in a Patient With Severe Bone Defect: A Case Report

**DOI:** 10.7759/cureus.113189

**Published:** 2026-07-22

**Authors:** Naveen Kumar D, Akash Pradip Bera, Manojit Basak, Yohan Kang, In-Bo Kim, Won Y Shon

**Affiliations:** 1 Orthopedics, Bumin Hospital, Busan, KOR

**Keywords:** 3d printing, crowe type iv, crowe type-iv, custom triflange acetabular component, hip dysplasia, patient-specific implant, total hip arthroplasty

## Abstract

A 67-year-old woman with Crowe type IV dysplasia of the right hip, severe acetabular bone loss, and an excessively narrow femoral canal secondary to childhood hip disease underwent hip arthroplasty using a custom triflange acetabular component and a custom-made femoral stem. Herein, we report a rare case of the use of both a custom-made acetabular component and a femoral component in high-grade hip dysplasia with a severe bone defect. Reconstruction of acetabular bone loss and an excessively narrow femoral stem is challenging. Using custom-made acetabular and femoral implants may improve radiological and functional outcomes.

## Introduction

Patients with childhood hip disorders (CHDs) and developmental dysplasia of the hip (DDH), particularly those with high-riding hip dislocations, often present with bone defects in both the acetabulum and proximal femur [1,2]. In Crowe type IV DDH, the acetabulum is characterized by a small, shallow cavity with deficient superolateral acetabular bone stock [3], making it difficult to achieve adequate coverage and stability with standard hemispherical cups [4]. Although acetabular bone defects can be managed using several reconstruction techniques [5], total hip arthroplasty (THA) in severe dysplastic hips remains technically challenging and is often associated with unsatisfactory outcomes. In extremely severe cases, THA may not be feasible [6,7]. Recent studies indicate good surgical outcomes after the use of customized acetabular cups in patients with DDH with acetabular bone defects [8,9]. However, most of these implants are either custom augments or cups with iliac flanges aimed at improving acetabular cup fixation rather than reconstructing the underlying acetabular bone defect [10-12].

The proximal femur in CHD and DDH often exhibits severe anatomical deformation, making standard off-the-shelf stems difficult to use [13]. Surgical techniques such as subtrochanteric osteotomy can sometimes facilitate conventional stem use but carry an increased risk of intraoperative and postoperative fractures [14,15]. In extreme cases, femoral splitting may be required [16].

Literature on THA using custom-made femoral stems in severely deformed proximal femurs remains scarce [17-19]. To our knowledge, no study has specifically reported the use of a custom stem in cases where the femoral canal was too narrow and the femur too slender to accommodate any standard implant, regardless of stem geometry or offset options. This represents a distinct clinical scenario, as narrow femoral canals pose unique challenges related to stem sizing and fit.

No part of this work has been previously presented at any conference, meeting, or symposia, nor has it been published as an abstract or poster.

## Case presentation

A 67-year-old woman presented with difficulty in walking due to severe right hip pain for the last three years. She had seen many orthopedic surgeons who had advised against surgery due to unsatisfactory acetabular and femoral architecture. She was ambulatory with support before the onset of symptoms. She was diagnosed with hyperlipidemia for which she was already undergoing treatment.

On examination, the patient was of average build, with right lower-limb shortening, inability to walk, and wasting of the right upper thigh muscles. The skin over the right hip was normal. Right hip range of motion was restricted due to pain. The true limb-length discrepancy (LLD) was approximately 4 cm. She also had a pre-existing fixed thoracolumbar kyphoscoliosis. As the patient was wheelchair-bound, gait assessment and the Trendelenburg test could not be performed. A fixed flexion deformity of 10° was noted at the right hip, along with an adduction contracture in the coronal plane (abduction 20°, adduction 5°) and a marked restriction of both internal and external rotation, which were negligible. Abductor muscle strength was reduced, likely secondary to chronic disuse atrophy. Pelvic obliquity was present, with elevation of the left hemipelvis, attributable to the combined effects of the hip contracture and the coexisting spinal deformity.

**Figure 1:**
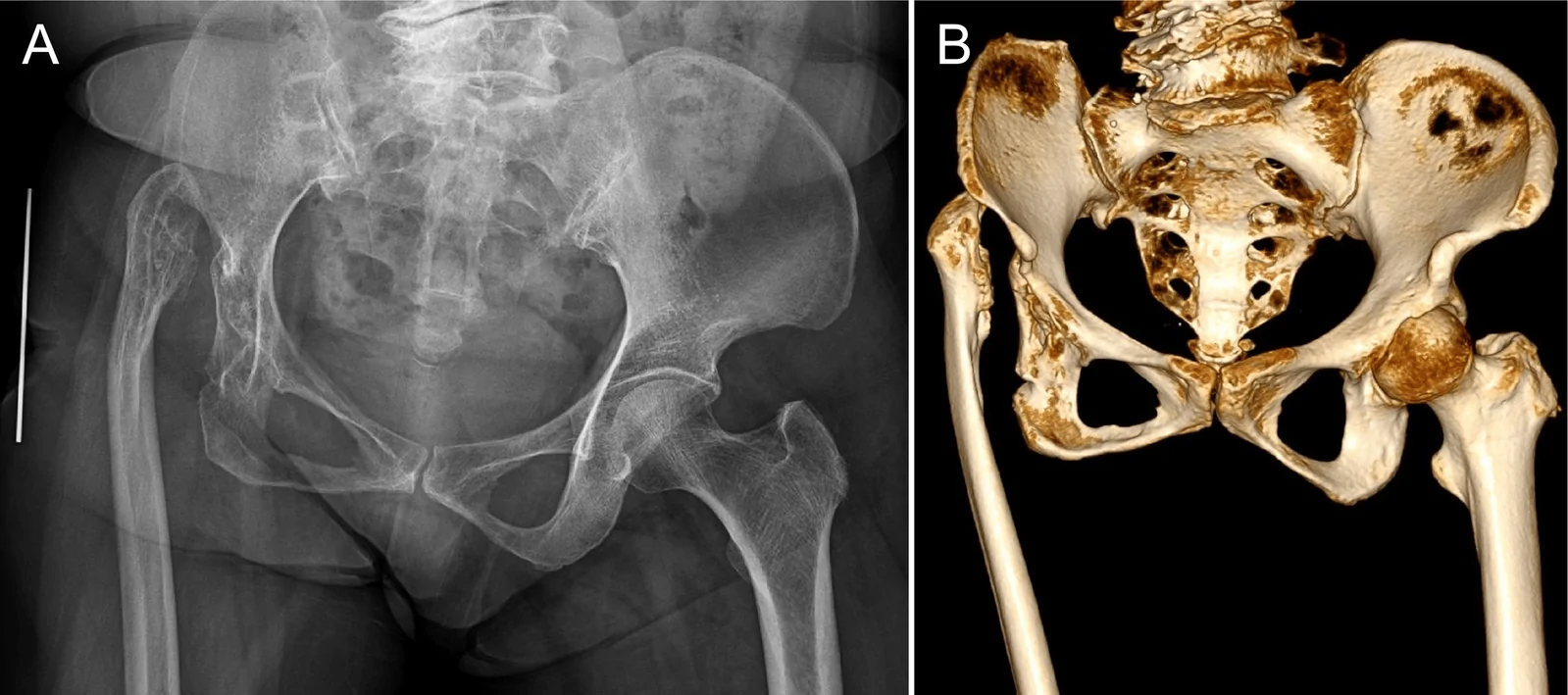
(A) Anteroposterior radiograph of the pelvis, demonstrating a Crowe type IV right hip dysplasia. (B) Three-dimensional computed tomography (3D-CT) reconstruction, demonstrating severe acetabulum bone defect and a narrow femoral canal.

Anteroposterior radiography of the pelvis depicted a shallow hypoplastic acetabulum and a narrow and distorted femoral canal (Figure 1A). Three-dimensional computed tomography (3D-CT) of the hip identified a small, true, distorted acetabulum with severe bone defect and deficient walls and a markedly narrowed femoral canal with reduced metaphyseal diameter (Figure 1B). The Harris Hip Score (HHS) [20] at presentation was 49. Written informed consent was obtained for THA using custom-made acetabular and femoral components and publication of this case.

A custom triflange acetabular component (CTAC) (Medyssey Co., Ltd., Seoul, Republic of Korea) was designed using bilateral 0.5 mm slice hip CT data, referencing the contralateral anatomy. Using 3D planning software (3-matic, Materialise NV, Leuven, Belgium), preoperative goals were set to restore the center of rotation (COR), targeting an alignment of 45° of inclination and 30° of anteversion. Given the patient's coexisting fixed thoracolumbar kyphoscoliosis, preoperative spinopelvic parameters were assessed, and the patient was classified as Hip-Spine Classification type 2B [20]. This classification guided the selected inclination and anteversion targets and supported the use of a dual-mobility (DM) construct, given the recognized increased dislocation risk associated with spinal stiffness and pathology in this population.

**Figure 2:**
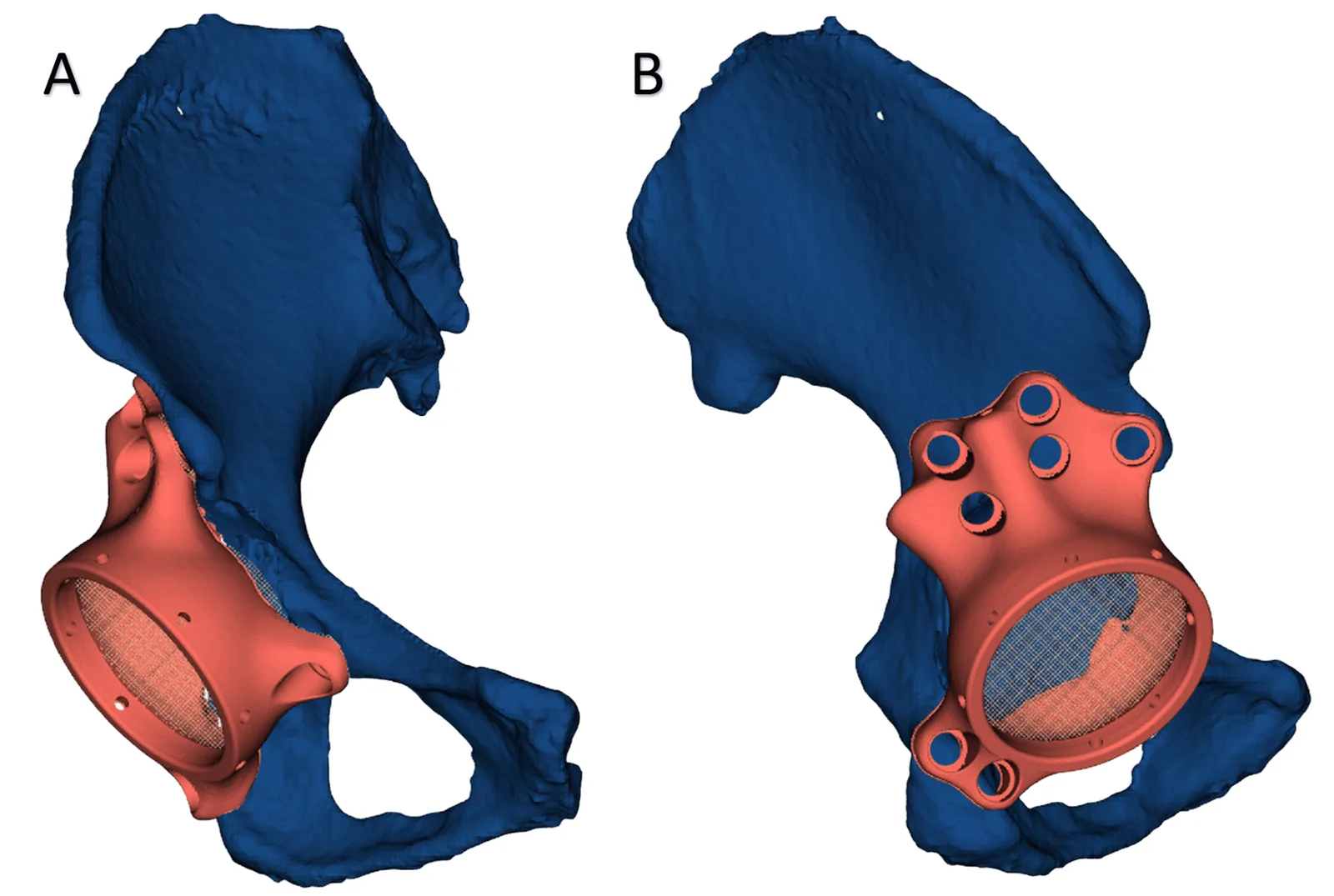
(A) Anterior and (B) lateral views of the custom triflange acetabular component (CTAC).

The implant was designed as a triflange acetabular component with a hemispherical cup and multiple flanges to achieve stable fixation. Screw trajectories and positions were planned according to the available bone stock (Figure 2). A predominantly solid structure was selected to enhance mechanical strength, with porous features incorporated where appropriate to facilitate osseointegration. The component was manufactured by Medyssey Co., Ltd. (Seoul, Republic of Korea) using Ti-6Al-4V ELI titanium alloy (ASTM F3001). Additive manufacturing was performed using electron beam melting (Arcam Q10 Plus, Arcam AB, Mölndal, Sweden), followed by standard post-processing and sterilization.

**Figure 3:**
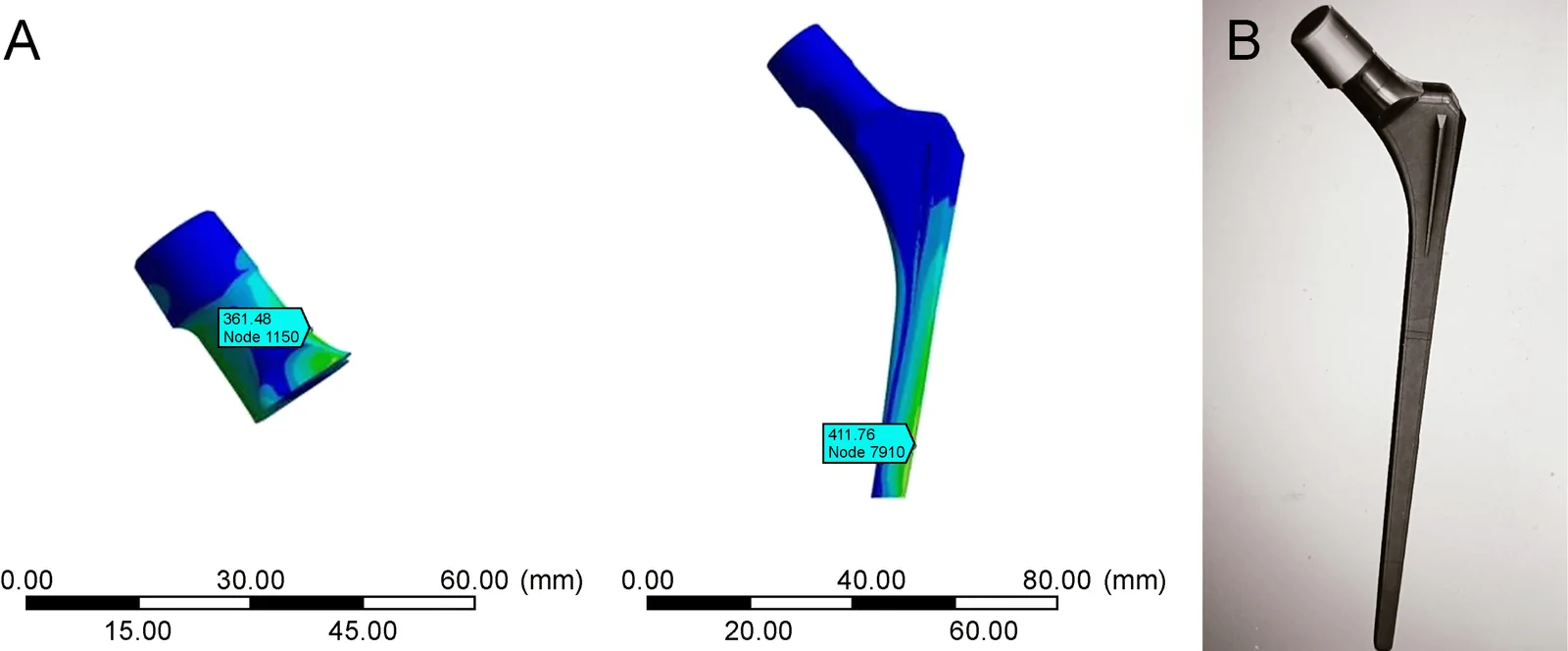
(A) Finite element analysis results of custom-made femoral stem according to ISO 7206-6 (left) and ISO 7206-4 (right). (B) The final design of the custom-made femoral stem.

A customized femoral stem was manufactured by Corentec Co., Ltd. (Cheonan, Republic of Korea) using Ti-6Al-4V ELI (ASTM F136), based on the commercially available Bencox® II cementless system (Corentec Co., Ltd., Cheonan, Republic of Korea). Preoperative radiographs were used for templating, using the smallest available stem as a reference. To improve fixation in the narrow canal, the stem length was increased with reduction of mediolateral thickness and increase of anteroposterior thickness to maintain structural strength. The component featured a 135° neck-shaft angle, a 28-mm offset, and a 12/14 trunnion for a 22-mm head. The design was converted into a 3D model, and finite element analysis performed using ANSYS (Ansys, Inc., Canonsburg, PA, USA) according to ISO 7206 standards indicated lower maximum Von Mises stress compared with the reference stem (Figure 3A). The implant was manufactured using conventional machining, followed by grit blasting to enhance osseointegration, and subsequent post-processing and sterilization (Figure 3B). The mechanical performance of six stems was verified via fatigue testing in accordance with ISO 7206-4. A slightly undersized customized femoral rasp was used for canal preparation and intraoperative trials. Both custom-made implants were certified by the Korean Ministry of Food and Drug Safety.

All the surgeries were performed by a senior arthroplasty surgeon. With the patient in lateral decubitus position under spinal anesthesia, a posterolateral incision was made along the greater trochanter to expose the acetabulum, ilium, ischium, and posterior column. Intraoperatively, the abductor musculature was noted to be markedly atrophic, consistent with chronic disuse. First, the proximal femur medullary cavity was exposed, then reaming and rasping were performed using the customized rasp. Transverse subtrochanteric osteotomy was performed 2 cm below the distal tip of the lesser trochanter. The osteotomized proximal femur was retracted proximally. The true acetabulum was identified by placing a retractor at the level of the teardrop, and exposure was extended proximally to the superior half of the ilium, anteriorly to the inferior iliac spine, and posteriorly to the sciatic notch. 

The small, deformed true acetabulum was reamed in accordance with the preoperative plan alongside simulation on a 3D-printed pelvic model, with reaming depth and orientation confirmed intraoperatively using fluoroscopic imaging. A trial plastic acetabular component was inserted, and precise placement was confirmed after burring and trimming with a high-speed burr. Following verification of accurate fitting in the 3D model and to the iliac, ischial, and pubic flanges, the CTAC was implanted and impacted to achieve stable fixation. Four cancellous locking screws (sizes 20 mm, 25 mm, 25 mm, and 25 mm) were inserted sequentially into the ischium, ilium, and pubis. A 46-mm cementless DM cup (Stryker Orthopaedics, Mahwah, NJ, USA) was cemented within the CTAC with 45° of inclination and 30° of anteversion.

After release of the iliopsoas and rectus femoris, a trial femoral rasp with a DM head and poly insert was fitted into the proximal fragment. It was reduced into the prosthetic cup while simultaneously monitoring tension on the sciatic nerve. Following trial head reduction, a second shortening osteotomy was done after the appropriate length of the subtrochanteric shortening was established based on the overlap between the fragments. The proximal femur trial stem was then reduced into the distal femur segment. Hip range of motion and stability were checked and deemed satisfactory. Proximal and distal fragments were wired to prevent peri-prosthetic fractures. The greater trochanter remained intact throughout the procedure and did not require separate stabilization. The custom-made femoral component was implanted with approximately 25° anteversion, after which stability and range of motion were assessed. A 13-cm structural on-lay allograft was secured with titanium cerclage wires at the osteotomy site to provide additional mechanical and biological support. 

**Figure 4:**
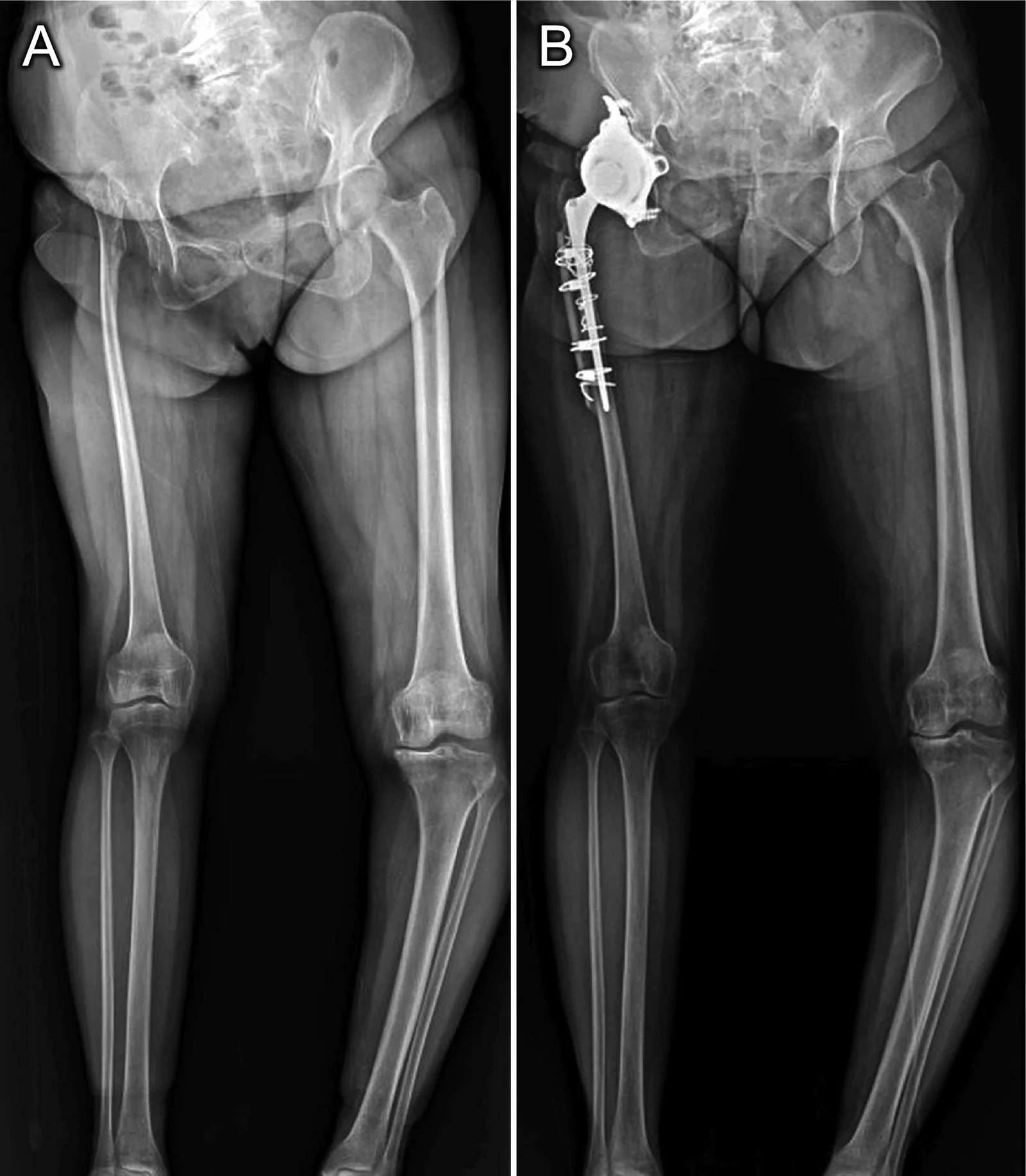
Anteroposterior full-length imaging of the lower extremity. (A) Preoperative showing pelvic tilt and a narrow femoral canal and (B) at two-year postoperative follow-up demonstrating pelvic tilt correction and restored limb alignment.

Postoperative radiographs confirmed anatomical restoration of the COR and a reduction in radiologic LLD to 0.5 cm (Figure 4). The patient was hospitalized for two weeks postoperatively. During this period, an acute posterior dislocation occurred following a sonographic assessment performed to evaluate the inflammatory status of the joint. After closed reduction failed, an open reduction was performed.

**Figure 5:**
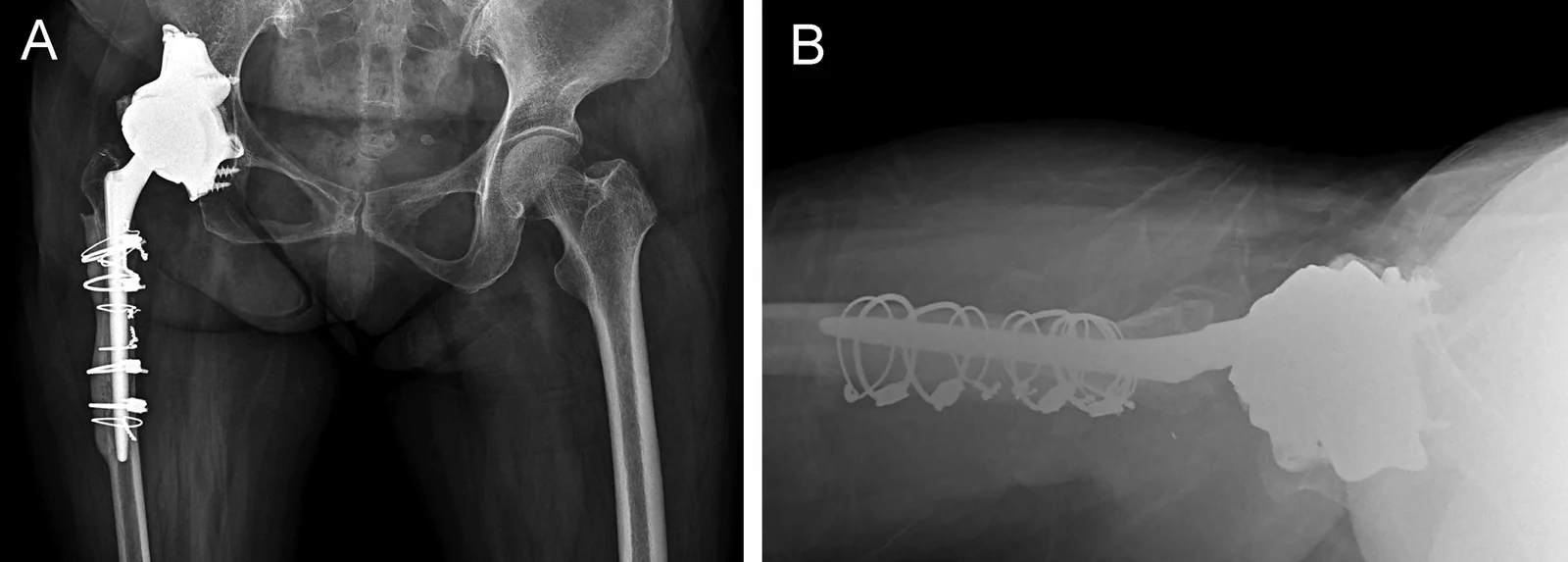
(A) Anteroposterior and (B) lateral imaging of the pelvis at the two-year follow-up, demonstrating restoration of the anatomical center of rotation and stable implant fixation.

An abduction brace was applied for five months, along with hip muscle strengthening exercises. Two weeks later, partial weight-bearing with crutches was allowed for three months. The patient was followed up clinically and radiographically at regular intervals of one month for six months, and then quarterly. At the two-year follow-up, the patient could walk painlessly with support. Successive imaging indicated no acetabular component loosening or femoral stem subsidence (Figure 5). The HHS [21] improved from 49 on the initial visit to 80 on the last visit.

## Discussion

This case demonstrates that anatomic reconstruction of a dysplastic hip using custom-made acetabular and femoral components can restore near-normal biomechanics with radiographic stability and meaningful functional gains. At two-year follow-up, clinical outcomes were favorable, with the patient reporting a high degree of satisfaction with the surgical result. Notably, however, THA using custom-made implants is associated with high rates of complications including infection, dislocation, and re-operation [21-23].

Despite using a DM cup within the CTAC (cup-in-cup technique) [24], with an acceptable range of component position, dislocation occurred in our case. Though primary THA with a DM cup is associated with a very low risk of dislocation [25-28], a 10.4% dislocation rate was reported after revision THA [29]. Solarino et al. [24] also reported that postoperative dislocation occurred in 3/16 patients undergoing DM THA with CTAC [30].

In our patient, chronic hip abductor and periarticular muscle atrophy, exacerbated by three years of wheelchair dependency, could have been the primary cause of instability [31]. Furthermore, compromised soft-tissue condition around the hip after childhood hip surgery precluded effective posterior soft-tissue repair. Lastly, sagittal spinal deformity and lumbar stiffness secondary to long-standing LLD further increased the dislocation risk by compromising spinopelvic balance [20]. Fortunately, there was no recurrence of dislocation thereafter [32].

Although customized implants are expensive and require extended production time, compared to the cost of managing early failures and revisions associated with standard implants or the debilitating pain the patient endures, the approach remains cost-effective [33]. However, when a surgeon encounters anatomical variations or poor bone quality, the options available to achieve primary stability become limited [23], and future long-term follow-up is needed to assess the survivorship of the new components.

## Conclusions

This case illustrates that combined custom-made acetabular and femoral components can achieve durable anatomical reconstruction and meaningful functional recovery in a primary THA setting where severe, concurrent acetabular and femoral bone deficiency renders standard implants unusable. Despite the occurrence of an early postoperative dislocation, the patient achieved restoration of the center of rotation, correction of limb-length discrepancy, and a marked improvement in HHS with stable, well-fixed implants at two-year follow-up. These findings suggest that patient-specific implants are a viable strategy for patients with altered bone morphology who have been refused conventional surgery, though careful attention to soft-tissue balance, abductor competence, and spinopelvic alignment is warranted to mitigate the elevated dislocation risk inherent to this population. Further studies with larger cohorts and longer follow-up are needed to confirm the long-term reliability of this dual-custom-implant approach.
